# Endothelial γ-Glutamyltransferase Contributes to the Vasorelaxant Effect of *S*-Nitrosoglutathione in Rat Aorta

**DOI:** 10.1371/journal.pone.0043190

**Published:** 2012-09-11

**Authors:** Fatima Dahboul, Pierre Leroy, Katy Maguin Gate, Ariane Boudier, Caroline Gaucher, Patrick Liminana, Isabelle Lartaud, Alfonso Pompella, Caroline Perrin-Sarrado

**Affiliations:** EA3452 CITHEFOR “Drug targets, formulation and preclinical assessment”, Faculté de Pharmacie, Université de Lorraine, Nancy, France; Max-Delbrück Center for Molecular Medicine (MDC), Germany

## Abstract

*S*-nitrosoglutathione (GSNO) involved in storage and transport of nitric oxide (^•^NO) plays an important role in vascular homeostasis. Breakdown of GSNO can be catalyzed by γ-glutamyltransferase (GGT). We investigated whether vascular GGT influences the vasorelaxant effect of GSNO in isolated rat aorta. Histochemical localization of GGT and measurement of its activity were performed by using chromogenic substrates in sections and in aorta homogenates, respectively. The role of GGT in GSNO metabolism was evaluated by measuring GSNO consumption rate (absorbance decay at 334 nm), ^•^NO release was visualized and quantified with the fluorescent probe 4,5-diaminofluorescein diacetate. The vasorelaxant effect of GSNO was assayed using isolated rat aortic rings (in the presence or absence of endothelium). The role of GGT was assessed by stimulating enzyme activity with cosubstrate glycylglycine, as well as using two independent inhibitors, competitive serine borate complex and non-competitive acivicin. Specific GGT activity was histochemically localized in the endothelium. Consumption of GSNO and release of free ^•^NO decreased and increased in presence of serine borate complex and glycylglycine, respectively. In vasorelaxation experiments with endothelium-intact aorta, the half maximal effective concentration of GSNO (EC50 = 3.2±0.5.10^−7^ M) increased in the presence of the two distinct GGT inhibitors, serine borate complex (1.6±0.2.10^−6^ M) and acivicin (8.3±0.6.10^−7^ M), while it decreased with glycylglycine (4.7±0.9.10^−8^ M). In endothelium-denuded aorta, EC_50_ for GSNO alone increased to 2.3±0.3.10^−6^ M, with no change in the presence of serine borate complex. These data demonstrate the important role of endothelial GGT activity in mediating the vasorelaxant effect of GSNO in rat aorta under physiological conditions. Because therapeutic treatments based on GSNO are presently under development, this endothelium-dependent mechanism involved in the vascular effects of GSNO should be taken into account in a pharmacological perspective.

## Introduction

Nitric oxide (^•^NO) is involved in several vascular functions, including smooth muscle cells relaxation and proliferation, platelet aggregation and leucocyte interaction with endothelium [1], [2]. In the vessel wall, ^•^NO released from endothelial cells diffuses to the smooth muscle cells layer and activates soluble guanylate cyclase to produce cyclic guanosine monophosphate, which in turn mediates smooth muscle cells relaxation [3]. Nitric oxide is highly reactive and has a short half life *in vivo*, estimated at a fraction of second.

*S*-nitrosoglutathione (GSNO) is an endogenous low molecular weight *S*-nitrosothiol, formed by nitrosation of reduced glutathione (GSH). *S*-nitrosoglutathione is involved in storage and transport of ^•^NO, exhibits higher stability than ^•^NO, mediates protein *S*-nitrosation processes and is thought to play an important role in vascular signaling [4]. The biological activity of GSNO and particularly its vasorelaxant effect have been reported in *ex vivo* isolated vessel model [5], [6], and are directly linked to its decomposition resulting in the release of ^•^NO.

*S*-nitrosothiols are quite stable *in vitro* at 37°C and pH 7.4 [7] but they are degraded *in vivo*, especially by enzymatic activities. One of them, the γ-glutamyltransferase (GGT; EC 2.3.2.2.) involved in the metabolism of GSH [8] has also been shown to metabolize GSNO [9], [10]. *S*-nitrosoglutathione is labile in the reducing environment of cytosol, on the other hand in the extracellular space γ-glutamyltransferase specifically catalyzes its breakdown releasing its γ-glutamyl residue through a hydrolytic pathway (1), and transferring this residue to an acceptor such as glycylglycine, in a transpeptidation reaction (2):
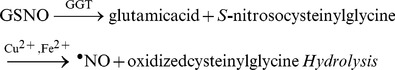
(1)

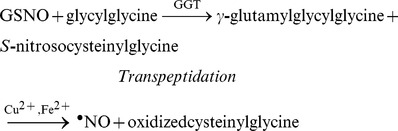
(2)


The presence of glycylglycine, added to serve as co-substrate for transpeptidation reaction [9], accelerates the decomposition rate of GSNO, thus producing *S*-nitrosocysteinylglycine more quickly. This latter being less stable than GSNO, it is rapidly decomposed in the presence of divalent metallic ions such as Fe^2+^ and Cu^2+^, leading to the release of ^•^NO and oxidized cysteinylglycine [9]. On the other hand, S-nitrosocysteinylglycine is itself provided with vasorelaxant activity, and the same is true for its metabolite S-nitroso-*L*-cysteine, produced by action of dipeptidases.

γ-Glutamyltransferase is highly expressed in endothelial cells [11] and therefore bioactivity of GSNO on the vessel wall might be affected by endothelial GGT activity.

To verify this hypothesis, we first measured the GGT activity in rat aorta and evaluated its role in the abitily of GSNO to release ^•^NO. Then, we determined the role of GGT in the vasorelaxant effect of GSNO, using the *ex vivo* model of isolated rat aortic rings with or without endothelium. Involvement of soluble guanylate cyclase in the relaxation induced by GSNO was also evaluated using ODQ as a specific inhibitor. In each experiment, the role of GGT was assessed by stimulating GGT activity with the exogenous γ-glutamyl acceptor glycylglycine, as well as by inhibiting it with competitive reversible inhibitor serine borate complex (SBC) [12], [13]. Non-competitive irreversible inhibitor acivicin was also employed in selected experiments [12], [13].

## Materials and Methods

### Chemicals and standards

All reagents were of analytical grade. 4,5-Diaminofluorescein (DAF-2) and 4,5-diaminofluorescein diacetate (DAF-2 DA) were obtained from Interchim (Montluçon, France). Acivicin, acetylcholine, L-N^G^-nitroarginine methyl ester (L-NAME), 1H- [1], [2], [4] oxadiazolo [4,3-a] quinoxalin-1-one (ODQ) and all other reagents were obtained from Sigma-Aldrich (Saint Quentin Fallavier, France). Ultrapure deionized water (>18.2 MΩ.cm) was used to prepare all solutions. Standard solutions of GSNO were prepared according to the method previously described [14]. The purity of GSNO was assessed by UV spectrophotometry using its molar absorbance at 334 nm (ε = 922 M^−1^.cm^−1^).

### Ethics Statement

All experiments were performed in accordance with the European Community guidelines (2010/63/EU) for the use of experimental animals, the respect of the 3 Rs' requirements for Animal Welfare (C. Perrin-Sarrado permit number n°54–72, French Ministry of Agriculture, Paris, France; protocols used in this study were described and approved by this permit).

### Preparation of aortic rings

Nine-week-old, male, normotensive, outbred Wistar rats (Janvier Laboratories, Le Genest St Isle, France; 480±32 g) were kept under standard conditions (temperature: 21±1°C, hygrometry 60±10%, lights on 6 am to 6 pm). Animals had free access to standard diet (A04, Safe, Villemoisson-sur-Orge, France) and water (reverse osmosis system, Culligan, Brussells, Belgium). After anesthesia (sodium pentobarbitone 60 mg.kg−1, i.p., Sanofi Sante Nutrition Animale, Libourne, France) and intravenous administration of heparin (500 units, heparine Choay), rats were sacrificed by exsanguination. A segment (3 cm) of the descending thoracic aorta was removed and immediately placed in ice-cold Krebs' solution containing 119 mM NaCl, 4.7 mM KCl, 1.2 mM KH_2_PO_4_, 1.2 mM MgSO_4_, 1.6 mM CaCl_2_, 24 mM NaHCO_3_, 5.5 mM glucose, adjusted to pH 7.4. Vessels were cleaned from surrounding connective tissues, cut in 5-mm long rings (4 rings per rat) and immediately used for vasoactivity studies. In some rings, the endothelium was removed by gently rubbing the intimal surface with forceps.

Some samples of aortic rings were immediately frozen in liquid nitrogen and stored at −80°C for subsequent biochemical studies. Additional samples were embedded in freezing medium (Tissue-Tek), frozen in liquid nitrogen and conserved at −20°C for histological/histochemical studies.

### Measurement of muscle tension

Aortic vasoactivity was measured as previously described [15], [16], isometric force transducer 0–10 g, Fort 10, World Precision Instruments, Sarasota, FL, USA). The bath was filled with Krebs' solution (1.5 mL, 37°C) and continuously bubbled with 95% O2 and 5% CO2. Following 60-min equilibration with a basal resting tension determined at 2 grams as giving the maximal response to α-adrenergic stimulation, rings were exposed 3 times to KCl (60 mM, 5 min, rings with a response lower than 4.5±0.5 g were discarded). Aortic rings (n = 6–12 per group, from 3–8 different rats in each group) were then precontracted with 1 µM phenylephrine. At the plateau of contraction, concentration-relaxation response curves to increasing concentrations of GSNO (0.001–10 µM) were performed. Endothelium integrity or removal was assessed by en face silver nitrate staining [17] and relaxation to 10 µM carbachol, with aortic rings showing less than 17±1% relaxation considered as endothelium-denuded [18].

The role of GGT in the vasorelaxant effect of GSNO was evaluated by comparing concentration-response curves in the absence and in the presence of either glycylglycine (20 mM, throughout the incubation with the different concentrations of GSNO), either SBC (20 mM, added since the start of the 60-min equilibration period) or acivicin (50 µM, added 60 min prior to relaxation experiments). Involvement of soluble guanylate cyclase in the relaxation induced by GSNO was evaluated using ODQ as a specific inhibitor (5 µM, added since the start of the 60-min equilibration period) [18]. Concentration-relaxation response curves to increasing concentrations of acetylcholine (0.001–10 µM) in the absence and in the presence of either 20 mM glycylglycine or 20 mM SBC were also performed as controls in order to check that both GGT modulators did not affect the vasorelaxation induced by a non-*S*-nitrosothiols ^•^NO donor.

### GGT activity, GSH and GSNO consumption in aorta homogenate

Aortic rings were homogenized in Tris buffer (100 mM, pH 7.4, Ultraturax® homogenizer, IKA, LaborTechnik, T25) and incubated for 2 h at 37°C in Tris buffer added with 1 mM of the synthetic GGT substrate L-γ-glutamyl-3-carboxy-4-nitroanilide (GCNA), GSH or GSNO. Where indicated, 20 mM glycylglycine or 20 mM SBC were added to the reaction medium. Every 20 min, 25 µL of reaction medium was withdrawn and centrifuged at 42,000 × g for 10 min at 4°C. Supernatants were used to determine (i) GGT activity and consumption rate of (ii) GSH and (iii) GSNO as follows (n = 3 in each condition):

- GGT activity was measured using GCNA according to Orlowski and Meister [19] in the presence of 10 mM MgCl_2_. Supernatant absorbance was read at 405 nm to monitor the release of 5-amino-2-nitrobenzoate (ε = 9500 M^−1^. cm^−1^) from GCNA;
- consumption rate of GSH in aorta homogenates was evaluated by measuring GSH concentration in supernatant at different times, using the fluorescence-based microtiter plate method previously described [20] based on the fluorescence intensity of the GSH-naphthalenedicarboxaldehyde adduct (λ_exc_  = 485±20 nm, λ_em_  = 528±20 nm, Synergy 2 model, Biotek Instruments, Colmar, France, calibration curve 0.65–3.25 µM);
- consumption rate of GSNO was calculated from the monitoring of the breakdown of S-nitrosated moieties (S-NO) in supernatant at 334 nm.

Protein concentrations were measured using the bicinchoninic acid method (Pierce Protein Assay Kit) with bovine serum albumin as standard. GGT specific activities, GSH and GSNO consumption rates are expressed in nmol of either obtained product or consumed substrate per min per mg of protein.

### Histochemical localization of GGT activity in aortic ring sections

Localization of enzymatically active GGT on tissue was performed with an azo-coupling reaction using γ-glutamyl-4-methoxy-2-naphthylamide (GMNA) as synthetic GGT substrate and Fast Garnet GBC as the chromogen [20]. Frozen aortic rings were cut into 8-µm thin sections (Cryostat Microm HM505E). Air-dried sections were incubated for 45 min at room temperature in freshly prepared reagent mixture containing 4 mM GMNA, 1 mM Fast Garnet GBC and 6 mM glycylglycine in PBS, pH 7.4, in the presence or absence of 20 mM SBC. Then slides were washed with tap water, air-dried and observed under light microscope with a 16× magnification. Negative controls were performed in the absence of substrate.

### Visualization and quantification of ^•^NO production in aortic rings

Visualization and quantification of ^•^NO production were operated using DAF-2 DA according to Rodriguez *et al*. with some modifications [21]. For ^•^NO quantification experiments, after 1-h preloading with 10 µM DAF-2 DA, aortic rings were rinsed then incubated for 15 min with 1 mM GSNO in the presence or absence of 20 mM SBC or 20 mM glycylglycine (n = 4–7 in each condition). To confirm that released ^•^NO was indeed deriving from added GSNO, control experiments were performed with eNOS inhibitor L-NAME, control incubations were performed in the presence of endothelial nitric oxide synthase (eNOS) inhibitor, 300 µM L-NAME [16], added 15 min before and throughout the 1-h DAF-2 DA preloading period. The subsequent nitrosated DAF-2 probe (DAF-2T) was extracted from the tissue using DMSO. Fluorescence intensity of diluted samples was read in a microplate reader (Biotek, USA, _λexc_  = 485±20 nm, _λem_  = 528±20 nm). Concentrations were evaluated using a calibration curve (0.025–0.250 µM) built with NaNO2 reacting with DAF-2.

For ^•^NO visualization, aortic rings preloaded (2 h) with the fluorescent probe were cut into 10-µm thick sections. Sections were then incubated (15 min, 37°C) with 1 mM GSNO in Krebs' solution, in the presence or absence of 20 mM SBC or 20 mM glycylglycine, mounted in suitable mounting medium (ProLong Antifade Kit, Invitrogen) and immediately observed with an inverted fluorescence microscope (Nikon TI-PS, _λexc_  = 480±20 nm, _λem_  = 527±15 nm). All images were captured under constant exposure time, gain and offset chosen to increase the threshold for fluorescence, thus attenuating background autofluorescence of elastin.

### Other procedures

All manipulations and assays involving GSNO were performed under conditions of subdued light, in order to minimize light-induced GSNO degradation.

### Data Analysis

The percentage of relaxation induced by GSNO (or carbachol) was calculated as:
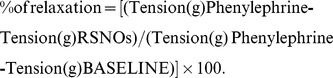



Half maximal effective concentration (EC_50_) was calculated using the logistic equation:
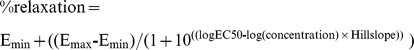
where E_min_ and E_max_  =  minimal and maximal response reached in each concentration-response curve. The value of Hill slope for all experiments was 0.9±0.1. Then – logEC_50_ values (pD_2_ values) were also calculated.

Results are expressed as means ± standard error of mean (SEM). For vasorelaxation studies, significant differences between groups were determined by a two-way ANOVA test (variables: endothelium and modulators of GGT activity) followed by a post-hoc Bonferroni test. For other experiments, a one-way ANOVA test followed by a post-hoc Bonferroni test was performed. The null hypothesis was rejected at p<0.05.

## Results

### Quantification and localization of GGT activity in aortic rings

Using the chromogenic substrate GCNA, aorta homogenates showed specific GGT activity (Table 1) which was abolished in the presence of SBC (values under limit of detection) and increased in the presence of glycylglycine. Correspondingly, using GSH as endogenous substrate of GGT, we showed that GSH consumption rate increased by 108±20% and decreased by 85±2% in the presence of glycylglycine and SBC, respectively (Table 1).

**Table 1:** Specific GGT activity and GSH consumption rate in rat aorta homogenates.

Conditions	GGT activity (nmol/min/mg proteins)	GSH consumption rate (nmol/min/mg proteins)	
**Control + glycylglycine**	0.30±0.01*	1.13±0.06*	
**Control**	0.12±0.01	0.55±0.02	
**Control + SBC**	ND^#^	0.08±0.01*	

Specific activity of γ-glutamyltransferase (GGT) and consumption rate of GSH measured in rat aorta homogenates incubated in Tris buffer pH 7.4 with and without SBC (20 mM) or glycylglycine (20 mM). Data are means ± S.E.M. of 3 experiments. (*p<0.05 versus Control; ND^#^: not detected – below limit of detection).

**Figure 1:**
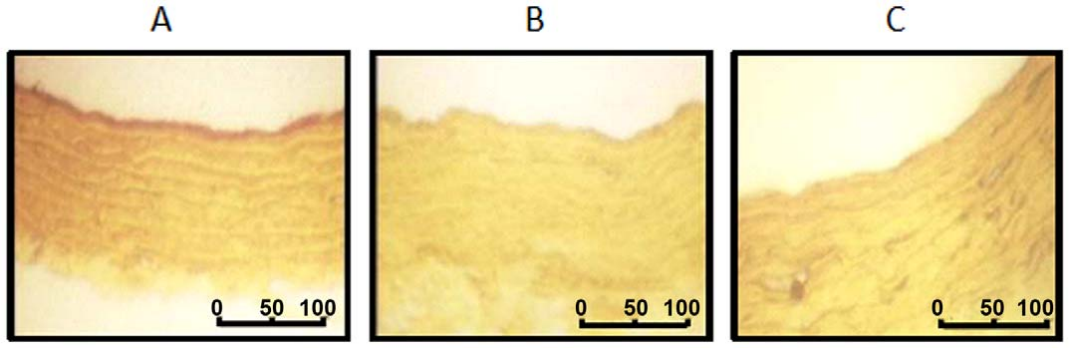
Histochemical localization of GGT activity in rat aortic tissue. Typical micrographs (×16) corresponding to histochemical visualization of γ-glutamyltransferase (GGT) activity in endothelium-intact rat aortic rings prepared in the presence of γ-glutamyl-4-methoxy-2-naphthylamide (GMNA, panel A) or in the presence of GMNA and SBC (20 mM, panel B) and in endothelium-denuded aortic rings prepared in the presence of GMNA (panel C). Scale bar, 100 µm.

GGT activity was histochemically detected in the endothelium (Figure 1A, pink to red staining) and some activity was also present in the layers neighboring the intima. Specificity was confirmed in endothelium-intact aortic rings incubated with SBC and in endothelium-denuded aortic rings, which both exhibited less intense staining (Figures 1B, 1C).

### GSNO consumption rate and GGT-dependent •NO release from GSNO in aorta

**Figure 2:**
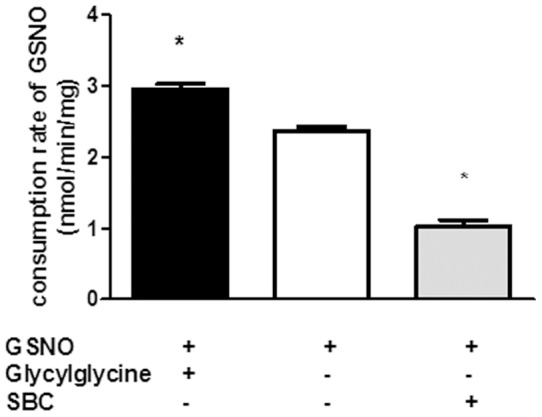
Effects of modulation of GGT activity on GSNO consumption in rat aorta homogenates. Consumption rates of GSNO measured in rat aorta homogenates incubated in Tris buffer pH 7.4 with and without SBC (20 mM) or glycylglycine (20 mM). Data are means ± SEM of 3 experiments, * p<0.05 *versus* substrate alone.

GSNO consumption was observed in aorta homogenates (2.4±0.2 nmol/min/mg proteins) (Figure 2) and was enhanced in the presence of glycylglycine by 24±4%, and reduced by 57±3% following the addition of SBC.

**Figure 3:**
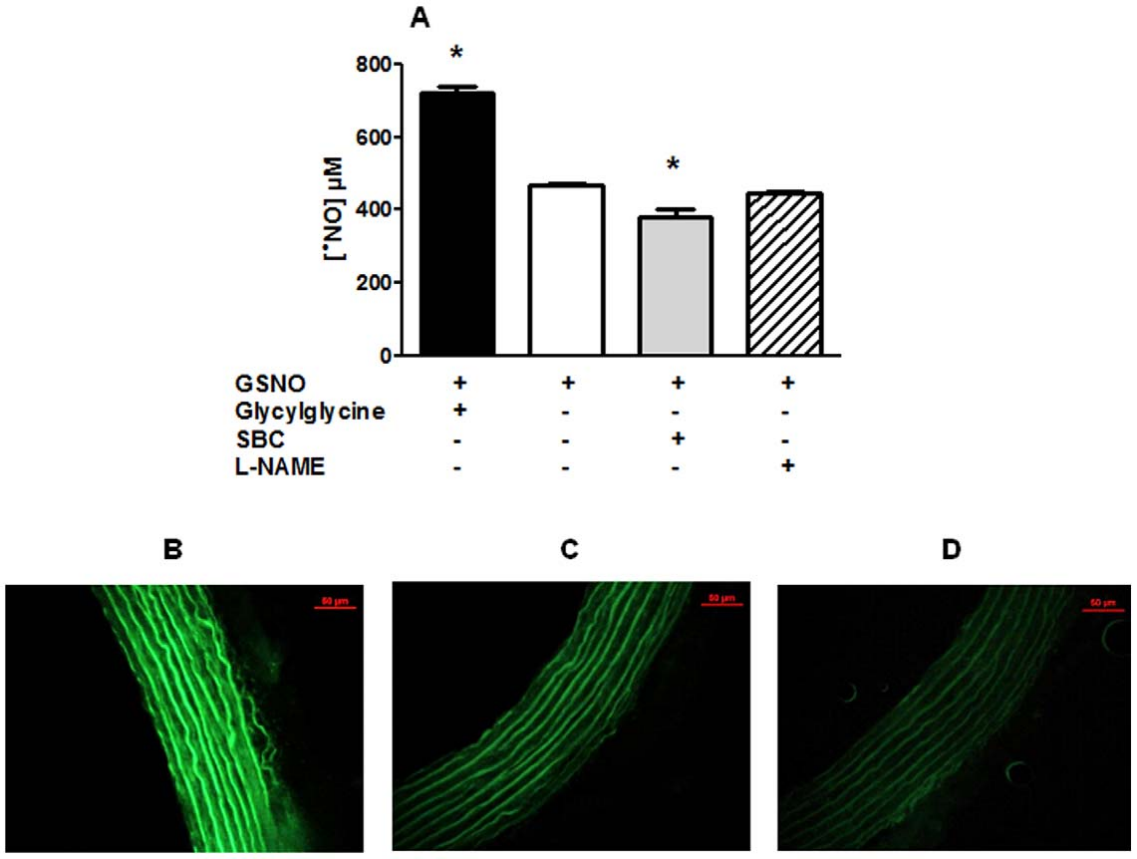
Effects of modulation of GGT activity on GSNO-mediated ^•^NO production in aortic tissue. Panel A: ^•^NO production from rat aortic rings exposed to GSNO (1 mM) or GSNO (1 mM) in the presence of SBC (20 mM), glycylglycine (20 mM) or L-NAME (300 µM). Data are means ± SEM of 4–7 experiments, * p<0.05 *versus* GSNO. Panels B, C, D: representative fluorescence micrographs (×20) of 10-µm sections of rat aortic rings loaded for 2 h with 4,5-diaminofluorescein diacetate (DAF-2 DA, 10 µM) showing ^•^NO production following 15-min incubation with GSNO (1 mM, panel C) or GSNO in the presence of SBC (20 mM, panel D) or glycylglycine (20 mM, panel B). Scale bar, 50 µm. All pictures were captured under constant exposure time, gain and offset.

Incubation of aortic rings with GSNO was associated with the release of •NO as quantified using the fluorescent probes DAF-2 DA. In the presence of SBC, •NO release was decreased by 20 % and strongly increased after GGT stimulation by glycylglycine (+ 45 %) (Figure 3A). Correspondingly, in aortic ring sections, DAF-2 DA revealed ^•^NO release following addition of GSNO (Figure 3C). The effect was diminished in the presence of SBC (Figure 3D) and increased in the presence of glycylglycine (Figure 3B).

Control experiments were carried out in order to verify whether glycylglycine or SBC might directly interact with GSNO and produce ^•^NO release. GSNO (1 mM) was incubated in 100 mM Tris buffer (pH 7.4, 37°C), alone or in the presence of glycylglycine (20 mM) or SBC (20 mM). No differences were appreciable in GSNO consumption in the three conditions studied during 2 h incubation time, as determined spectrophotometrically at 334 nm (*data not shown*).

Control experiments were also performed in order to verify the possibility that glycylglycine or SBC might directly interact with DAF-2 DA or affect tissue autofluorescence. Sections of aortic rings were thus incubated (15 min, 37°C) in Krebs solution in the presence of DAF-2DA without additions (control-basal) and after addition of SBC alone (control-SBC) or glycylglycine (control-glyglycine). Sections were then analysed by microscopy, as well as fluorescence was extracted from tissue with DMSO and analysed spectrofluorimetrically. Basal fluorescence was indeed appreciable in aortic rings, which was however not modified after incubation with SBC or with glycylglycine (*data not shown*), suggesting that the two compounds did not modify tissue autofluorescence nor did they exert effects on ^•^NO production independently of exogenously added GSNO.

Finally, to confirm that released ^•^NO was indeed deriving from added GSNO, control experiments were performed with eNOS inhibitor L-NAME. Indeed, eNOS inhibition did not modify the amount of ^•^NO released following the addition of GSNO (Figure 3A). Thus confirming that eNOS was not involved in the described phenomena.

### Vasorelaxant effects of RSNOs on isolated aortic rings

**Figure 4:**
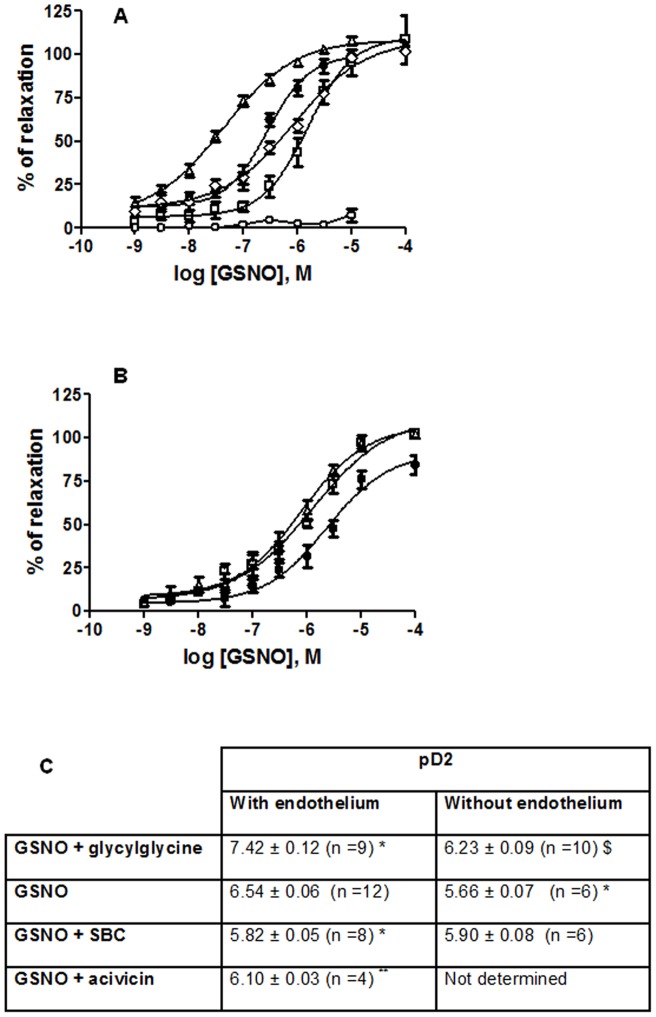
Influence of GGT activity on the vasorelaxant effect of GSNO. Concentration-dependent vasorelaxation response curves of GSNO (•), GSNO with glycylglycine (20 mM) (Δ), GSNO with acivicin (50 µM) (◊), GSNO with SBC (20 mM) (□), and GSNO with ODQ (5 µM) (○) in endothelium-intact (panel A) and endothelium-denuded (panel B) aortic rings. Panel C: pD2 values (panel C). Data are means ± SEM of 4–12 aortic rings per group. p values (2 ways ANOVA test): p_interaction_ <0.0001; p_endothelium_ <0.0001; p_modulator_ _of GGT_ <0.0001. * p<0.05 *versus* GSNO endothelium-intact; $ p<0.05 *versus* GSNO endothelium-denuded (post-hoc Bonferroni test). ** p<0.05 versus GSNO in endothelium-intact aortic rings (one way ANOVA test).

In endothelium-intact aortic rings precontracted with phenylephrine, GSNO induced a concentration-dependent relaxation (Figure 4A, EC_50_ = 3.2±0.5.10−7 M, pD2 panel C). In the presence of glycylglycine, the curve was shifted to the left, with a 7 times lower EC_50_ value (pD_2_ +1 log). On the opposite, in the presence of SBC or acivicin the curves were shifted to the right, EC_50_ was 5 or 3 times higher and pD2 decreased by 0.7 log or 0.5 log, respectively.

To verify if glycylglycine or SBC might directly affect aortic ring contraction independently of GGT activity, control experiments were performed using non-SNO agonist acetylcholine. Neither compound did modify acetylcholine-induced vasorelaxation (*data not shown*). Experiments were also performed using guanylate cyclase inhibitor ODQ, which suppressed GSNO-induced vasorelaxation (Figure 4A) and confirmed that the observed effect was indeed mediated through production of cGMP.

In endothelium-denuded aortic rings, all GSNO concentration-response curves were shifted to the right in comparison to endothelium-intact rings (pD2 -1 log), excepted with SBC which had no further effect (Figure 4B and panel C).

## Discussion

The aim of this study was to investigate the role of the endothelial GGT on the metabolism and vasorelaxant effect of GSNO on aorta in physiological conditions. We showed that GGT activity is present in endothelial cells, is involved in the metabolism of GSNO and in the subsequent release of •NO. This led to the vasorelaxant effect of GSNO which was improved in the presence of endothelium. The GGT activity thus plays a pivotal role in the endothelium-dependent vasorelaxant properties of GSNO.

Previous studies *in vitro* have biochemically characterized the metabolism of GSNO by GGT, showing that the affinity of GGT for this substrate is comparable to that of GSH [22]. However, few data are available about the importance of GGT activity in the bioactivity of GSNO in vascular tissue. In the present study, GGT activity was mainly localized in the endothelial layer as previously described [11]. This activity, measured in rat aorta homogenates using the specific chromogenic substrate GCNA, was comparable to values previously measured in human mammary artery tissue [23]. The specificity of the reaction was confirmed with SBC, a specific reversible GGT inhibitor, as well as with non-competitive inhibitor acivicin, and with glycylglycine, an acceptor of γ-glutamyl residue used for stimulation of GGT activity [24]. The 20 mM concentration of SBC was chosen on the basis of our previous work where it decreased more than 95% of GGT activity in cerebral microvessels, a tissue with rich GGT activity [20]. Twenty millimolar of glycylglycine was previously shown to accelerate GGT-dependent GSNO decomposition in rat kidney homogenates [10].

GGT activity was also evaluated with its endogenous substrate GSH, in the same conditions than with the synthetic substrate GCNA. As expected, SBC significantly decreased the consumption rate of GSH, whereas addition of glycylglycine strongly increased it.

The subsequent observations that SBC decreased and glycylglycine accelerated GSNO decomposition strongly indicate that GGT is responsible for GSNO breakdown in aorta homogenates. Moreover, GSNO induced the ^•^NO release, which was quantified and histochemically visualized in aorta with the fluorescent probe DAF-2 (Figure 3). This ^•^NO production was reduced in the presence of SBC and enhanced in the presence of glycylglycine. Altogether, our results indicate that GSNO (i) is metabolized in rat aorta and (ii) its breakdown, and the subsequent release of ^•^NO depend on GGT activity.

Inhibition of GGT resulted in only partial inhibition of GSNO consumption (Figure 2) which suggests that other enzyme activities are involved in GSNO metabolism in the aorta. This is in agreement with recent studies showing the role of other systems in *S*-nitrosothiols uptake and degradation [25], such as amino acid membrane transporters or protein disulfide isomerases [26], and thioredoxin [27], a protein with a redox active disulfide.

We demonstrate that GSNO induced a rapid and concentration-dependent relaxation in endothelium-intact precontracted aorta, in agreement with previously published studies [5], [26], [28], [29]. As shown by results obtained with specific inhibitor ODQ, this effect can be explained with the rapid activation of soluble guanylate cyclase following ^•^NO release, resulting in the fast relaxation of vascular smooth muscle cells. Our interesting results, showing increased or decreased EC_50_ of GSNO after inhibition or stimulation of GGT, respectively, strongly confirm the major role of GGT in the vasorelaxant effect of GSNO in the aorta. Moreover, removal of endothelium changed the impact on the vasorelaxant properties of GSNO. As expected, the effect of GSNO was decreased in the absence of endothelium (7-fold higher EC_50_). GSNO-induced vasorelaxation was similar in endothelium-intact/GGT inhibited (SBC) aortic rings and in endothelium-denuded aorta (pD_2_ values not different between the two conditions). These observations further confirm the role of endothelial GGT in the vasorelaxation induced by GSNO.

In endothelium-denuded rings, addition of glycylglycine surprisingly increased the pD_2_ value of GSNO. Removal of endothelium was mechanically performed and we cannot exclude that few endothelial cells and consequently some active GGT remained behind in some rings. The presence of transition metals (copper, iron) has been shown to to catalyze the GGT-mediated release of NO from GSNO [9], [10]. As no metals were exogenously added in our experimental systems, decomposition of RSNO was likely supported by metals present as contaminants in incubation media, as previously described in our laboratories [9].

To the best of our knowledge, only one study has previously investigated the implication of GGT in the rapid relaxation induced by GSNO and other RSNO's such as *S*-nitrosophytochelatins in rat aortic rings [26], but the specific role of endothelium was not evaluated.

In conclusion, our results show for the first time that, in physiological conditions, the rapid vasorelaxant effects of GSNO are largely dependent on the presence of endothelium and are mediated through its GGT-dependent metabolism. This is an important observation that should be taken into account in a pathophysiological perspective, because elevated GGT activity has been associated with various pathologies such as cystic fibrosis [30], hypertension [31] and atherosclerosis [23], and because therapeutics treatments based on GSNO have already been proposed at the clinical level [32], [33]. Future studies in animal models will help to clarify whether modulation of endothelial GGT activity can be used as a pharmacological tool in order to improve the effects of GSNO-based therapies.
